# Promoting rigor and sustainment in implementation science capacity building programs: A multi-method study

**DOI:** 10.1177/26334895221146261

**Published:** 2022-12-25

**Authors:** Amy G. Huebschmann, Shelly Johnston, Rachel Davis, Bethany M. Kwan, Elvin Geng, Debra Haire-Joshu, Brittney Sandler, Demetria M. McNeal, Ross C. Brownson, Borsika A. Rabin

**Affiliations:** 1 Adult and Child Center for Outcomes Research and Delivery Science, University of Colorado Anschutz Medical Campus (CU-Anschutz), Aurora, CO, USA; 2 Division of General Internal Medicine, CU-Anschutz, Aurora, CO, USA; 3 Ludeman Family Center for Women’s Health Research, CU-Anschutz, Aurora, CO, USA; 4 Washington University Center for Diabetes Translation Research, 7548 Washington University in St. Louis, St. Louis, MO, USA; 5 Health Service and Population Research Department, Centre for Implementation Science, 4616 King’s College London, London, UK; 6 Department of Emergency Medicine, CU-Anschutz, Aurora, CO, USA; 7 Colorado Clinical & Translational Sciences Institute, CU-Anschutz, Aurora, CO, USA; 8 Department of Medicine (Division of Infectious Diseases), Washington University School of Medicine, 7548 Washington University in St. Louis, St. Louis, MO, USA; 9 Department of Medicine (Division of Geriatrics and Nutritional Sciences), Washington University School of Medicine, 7548 Washington University in St. Louis, St. Louis, MO, USA; 10 Prevention Research Center, Brown School at Washington University, St. Louis, MO, USA; 11 Department of Surgery (Division of Public Health Sciences) and Alvin J. Siteman Cancer Center, Washington University School of Medicine, Washington University, St. Louis, MO, USA; 12 ACTRI Dissemination and Implementation Science Center, University of California San Diego, La Jolla, CA, USA; 13 Herbert Wertheim School of Public Health and Human Longevity Science, University of California San Diego, La Jolla, CA, USA

**Keywords:** implementation, training, equity, building, research competency, curriculum

## Abstract

**Background:**

The field of Implementation science (IS) continues to evolve, and the number and type of IS capacity building Programs (ISCBPs) are in flux. These changes push the field to revisit the accepted IS competencies and to guide sustainment of ISCBPs. Our objectives were: (1) compare characteristics of current ISCBPs; (2) identify recommendations to support ISCBP sustainment; (3) measure how often ISCBPs address IS competencies; (4) identify novel and important IS competencies for the field.

**Method:**

This multi-method study included ISCBPs delivering structured, longitudinal IS training, excluding single courses and brief workshops. We used three complementary methods to meet our objectives. First, we identified ISCBPs via an internet search and snowball sampling methods. Second, we surveyed these ISCBPs to identify areas of program focus, types of trainees, IS competencies addressed, and recommendations to sustain ISCBPs. Third, we conducted a modified Delphi process with IS researchers/leaders to reach consensus on the IS competencies that were both important and novel as compared to the IS competencies published to date.

**Results:**

Among 74 eligible ISCBPs identified, 46 responded (62% response rate). Respondent ISCBPs represented diverse areas of focus (e.g., global health, cardiopulmonary disease) and trainee stages (e.g., graduate students, mid-career faculty). While most respondent ISCBPs addressed core IS methods, targeting IS competencies was less consistent (33% for nongraduate/non-fellowship ISCBPs; >90% for graduate/national ISCBPs). Our modified Delphi process identified eight novel and important IS competencies related to increasing health equity or the speed of translation. Recommendations to sustain ISCBPs included securing financial administrative support.

**Conclusions:**

Current ISCBPs train learners across varying career stages in diverse focus areas. To promote rigor, we recommend ISCBPs address specific IS competencies, with consideration of these eight novel/emerging competencies. We also recommend ISCBPs report on their IS competencies, focus area(s), and trainee characteristics. ISCBP programs need administrative financial support.

**Plain Language Summary:**

There is a limited workforce capacity to conduct implementation science (IS) research. To address this gap, the number and type of IS capacity building Programs (ISCBPs) focusing on training researchers and practitioners in IS methods continue to increase. Our efforts to comprehensively identify and describe ISCBPs for researchers and practitioners highlighted four implications for leaders of ISCBPs related to program sustainment and rigor. First, we identified a range of contextual characteristics of ISCBPs, including the research topics, methods, and IS competencies addressed, and the types of trainees accepted. Second, given the variability of trainee types and research, rigorous ISCBP programs should tailor the IS competencies and methods addressed to the skills needed by the types of trainees in their program. Third, the field of IS needs to periodically revisit the competencies needed with attention to the skills needed in the field. We used a consensus-building process with ISCBP leaders and other IS experts to expand existing IS competencies and identified eight important, novel IS competencies that broadly relate to promoting health equity and speeding the translation of research to practice. Finally, as more institutions consider developing ISCBPs, we identified factors needed to support ISCBP sustainment, including ongoing financial support. In addition to these implications for ISCBP leaders, there are also policy implications. For example, IS journals may enact policies to require manuscripts evaluating ISCBP performance to report on certain contextual characteristics, such as the IS competencies addressed and types of trainees accepted. The field may also consider developing an accreditation body to evaluate the rigor of ISCBP curricula.

## Introduction

There are substantial delays in the implementation of diverse clinical and public health interventions (Balas & Boren, 2000; Green et al., 2009; Khan et al., 2021; Lee, 2007). Spurred on by this gap between research and practice, implementation science (IS) has emerged as a crosscutting, transdisciplinary, and rapidly developing area of science (Chambers, 2018; Eccles & Mittman, 2006; Khan et al., 2021; Kwan et al., 2022). The field of IS seeks to translate evidence-based programs and policies (EBPPs) into routine care and community practice in a timely fashion, in order to improve public health and healthcare delivery.

To leverage the benefit of IS for improving health, IS capacity building programs (ISCBPs) have been developed to train researchers to develop and test EBPPs with attention to context, and to train IS practitioners to competently implement and disseminate these EBPPs (Brownson, Jacob, et al., 2021; Davis & D'Lima, 2020; Leppin et al., 2021; Norton, 2014; Proctor & Chambers, 2017). However, due in part to the rapid evolution of IS as a scientific discipline, there has been a persistent under-supply of IS experts and ISCBPs, as compared to the high demand among researchers and practitioners to receive training in IS methods (Brownson et al., 2018). Unfortunately, many national ISCBPs can only accept ≤20% of applicants, due to limited slots for trainees (Davis & D'Lima, 2020; Kho et al., 2009; Vinson et al., 2019). In response to this need, there has been a burgeoning development of ISCBPs by academic institutions, national funding agencies, and other groups.

This creates a challenge for those leading current ISCBPs or seeking to develop new ISCBPs to meet this demand for training, both in terms of the rigor of the training delivered, and in terms of ensuring there are sufficient resources to sustain a program. For example, in terms of ensuring sufficient rigor of training, several reviews have recommended ISCBPs to address a gold standard set of IS competencies (Brownson, Jacob, et al., 2021; Padek et al., 2015), such as appropriate selection of IS frameworks, models, and theories. The initial set of 43 core IS competencies developed by Padek and colleagues are organized into three levels of expertise (i.e., beginner, intermediate, advanced), and four broad domains: 1. Definition, Background and Rationale; 2. Theory and Approaches; 3. Design and Analysis; 4. Practice-based considerations (Padek et al., 2015). To date, these are considered as the “gold standard” competencies for IS (Brownson, Jacob, et al., 2021; Padek et al., 2015). As reported previously, examples of these IS competencies include: appropriate selection of IS frameworks, models, and theories; designing and testing implementation and dissemination strategies (i.e., interventions at the setting level); determining appropriate pragmatic study designs, such as hybrid trials for testing effectiveness and implementation); processes for guiding iterative adaptation; and measurement of pragmatic outcomes, such as implementation and sustainability (Gonzales et al., 2012; Padek et al., 2015; Schultes et al., 2021).

Recent calls for ISCBPs to include IS competencies in their curriculum and evaluation process clarify the need for attention to rigor (Proctor & Chambers, 2017). In addition, there are general recommendations to tailor training to the specific needs of trainees, and to report on the types of IS competencies addressed and trainees served (Proctor & Chambers, 2017)—however, there have not been any consistent accreditation standards to follow. The recent influx of new ISCBPs and sunsetting of prior ISCBPs highlights a particular need to identify any gaps in the current programmatic focus and to consider program characteristics related to sustainment. Recommendations for the field of IS to expand its methodologic attention to health equity (Adsul et al., 2022; Brownson, Kumanyika, et al., 2021; Kwan et al., 2022; Leppin et al., 2021) and to differentiate training for implementation practitioners from implementation scientists (Leppin et al., 2021) are two examples of the need to periodically revisit competencies to ensure that they meet the needs of trainees. Informed by these needs in the literature and our collective experiences leading ISCBPs, we have identified several key questions to advance our understanding of ISCBPs and related IS competencies by conducting this multi-method study. This multi-method study included a scoping review that informed a survey of ISCBPs and a modified Delphi panel to build consensus on novel IS competencies.

Scoping reviews are defined as “exploratory projects that systematically map the literature available on a topic, identifying key concepts, theories, sources of evidence, and gaps in the research (Peters et al., 2021). Our rationale for use of scoping review methodology is the emerging nature of the data on ISCBPs. More specifically, few programs have published the competencies that they use or the resources that they require. Scoping reviews require identification of a target program (or population), a key focus, and the contextual influences. This review sought to identify contextual influences on the key foci of sustainment and rigor among the target programs of ISCBPs (Peters et al., 2021). Our multi-method study aims included: Aim 1. Contextual influences: what range of characteristics of ISCBPs exist in terms of trainee type, program resources/scope, research methods focus, research content area, and IS competencies addressed? Aim 2. Sustainment: what recommendations do ISCBPs have to promote their sustainment? Aim 3. Rigor: how often are ISCBPs addressing IS competencies in their curriculum, and what competencies do they currently address? Aim 4. Novel competencies/training needs: are there novel IS competencies that ISCBPs should address? These aims are relevant both to current leaders of ISCBP programs seeking to sustain a rigorous program, as well as to individuals seeking to find rigorous ISCBP programs with strong potential for sustainment.

## Method

### Brief Overview of Multi-Method Design

To accomplish the four key aims, this multi-method study included the following overarching approaches. Aims 1–3 sought to survey all current ISCBPs, not just those with published outcome data (Chambers et al., 2017; Davis & D'Lima, 2020; Proctor & Chambers, 2017). Aim 4 sought to identify novel/emerging IS competencies both from a scoping review of the published literature and from the needs identified by existing ISCBPs. The Aims 1–3 survey and Aim 4 competency revision processes received exempt status from the Colorado Multiple Institutional Review Board and the Washington University Institutional Review Board, respectively.

For Aims 1–3, we defined ISCBPs as a longitudinal, structured set of activities intended to provide training in IS methods to researchers, program evaluators, or implementation practitioners. A research assistant e-mailed a Qualtrics© survey link to program leaders of identified ISCBPs in July 2020 to obtain these data—two additional requests were e-mailed over 2 months to non-responding ISCBPs. The survey assessed several elements, including their training focus areas and any specific IS competencies addressed—see “Survey domains for Aims 1–3” below for details. Survey questions included both multiple-choice and open-ended answer response options, including an “Other” category with open-ended responses when multiple-choice responses were not applicable. For Aim 4, we sought to identify novel IS competencies as compared to the current gold standard for IS competencies (Padek et al., 2015), by extracting IS competencies through a three-step process of a literature review as well as the above-noted survey of IS competencies used by current ISCBP programs (Padek et al., 2015). We then used a modified Delphi approach to come to consensus on which of these novel competencies should be added to the current gold standard IS competency list. Based on these multiple methods, we synthesized the results of the Aims 1–3 survey of ISCBPs and the novel/emerging IS competencies from Aim 4 into recommendations for those seeking to develop and/or sustain an ISCBP. We provide further details on each aspect of these methods below.

### Justification for Search Strategies for Aims 1–3 and Aim 4

According to current guidance for scoping reviews, we provide justification for the databases searched and the search strategy used (Peters et al., 2021). There were two separate search strategies undertaken for this study, each with its own database. For our search strategy for Aims 1–3 to identify ISCBPs, we utilized the internet (Google©). Our justification for conducting this search on the internet rather than in SCOPUS or National Institutes of Health (NIH) Reporter is that many ISCBPs are not funded by research dollars and publications do not cite them, but ISCBPs typically use a website for recruitment of trainees. The content obtained for Aims 1–3 came from the surveys of the ISCBP respondents rather than from the internet. The search strategy for Aim 4 to identify IS competencies for ISCBPs to address was conducted in the SCOPUS database with supplementation from the competencies reported in Aim 3 by the ISCBPs identified via internet search. The rationale for use of this database for the Aim 4 search is that it includes peer-reviewed journals, book series, and abstracts.

### Implementation Science Capacity Building Program Identification for Aims 1–3

Three complementary approaches were used to identify ISCBPs: (1) internet search (Google©) on May 1, 2020 by a research assistant for current ISCBPs, using the following terms: (“implementation science” OR “implementation research” OR “D&I research”) AND (“training” OR “capacity building” OR “graduate”); (2) existing list of ISCBPs on the Society for Implementation Research Collaboration website; (3) snowball sampling process through referrals from leaders of the ISCBPs identified through the first two approaches for additional ISCBPs that were not yet included. This process identified potential ISCBPs for inclusion in the survey to ascertain data for Aims 1–3.

### Inclusion Criteria for Intensive ISCBP

ISCBPs were included if they could be classified in one of the following categories of moderately intense IS training that would warrant attention to the use and evaluation of competencies: ISCBP Category 1: training institutes delivered to a national/international group of trainees by a national or international sponsor to build IS research capacity in that region; ISCBP Category 2: mentored fellowship training programs focused on IS science competencies, typically offered by a specific academic institutional department or organization; ISCBP Category 3: academic graduate-level training programs with a sequence of at least three complementary courses in IS that provide a strong foundation to build IS competencies; ISCBP Category 4: other training programs, typically short-term but at least 2 days in duration, and offered by an academic institutional department.

### Survey Domains for Aims 1–3

For our survey assessment of Aims 1–3, the survey items were informed by the recommendations for ISCBP programs in recent reviews of IS capacity building and training (Davis & D'Lima, 2020; Proctor & Chambers, 2017). We use symbols to designate domains recommended by Davis & D’Lima (≠) and by Proctor and Chambers (*) in the following numbered list. Specifically, the survey assessed: (1) program type*≠; (2) target audience for the program*≠ (e.g., graduate student, postdoctoral fellow, early-career faculty); (3) specific areas of focus in terms of both research content area*≠ (e.g., cardiovascular disease, cancer, mental health) and research methods*≠; (4) average number of trainees accepted per year; (5) cost of the program to trainees, including whether trainee tuition is required ≠; (6) year when program was established≠; (7) any publications describing the program and/or its evaluation≠; (8) website for program≠; (9) the number of courses or credit hours required to complete (N/A—training institute)≠; (10) average length of time to complete the program≠; (11) IS competencies targeted by the program*≠; (12) status of the program (active/inactive/in development)*; (13) necessary program support for sustainability* (e.g., financial support for director and/or administrator, grant funding). The above items addressed Aims 1 and 3. To address Aim 2 regarding recommendations for sustainment, the survey included an open-ended question regarding lessons learned from ISCBP program development. Two coauthors (AGH and BAR) reviewed the program responses to the survey to confirm eligibility.

### Aim 4 Approach to Revise IS Competencies and Identify Novel Competencies

We sought to replicate past approaches used to develop standards for IS competency-based education (Padek et al., 2015). Specifically, this included a consensus-building process to identify IS competencies that researchers need to develop and that ISCBPs need to provide, and to designate these competencies as attainable for a relative beginner, intermediate, or advanced trainee (Padek et al., 2015).

To identify novel competencies as compared to the gold standard IS competencies (Padek et al., 2015), we undertook a three-step process to extract novel IS competencies from our Aim 4 literature review and from the Aim 3 competencies submitted from our survey of ISCBPs. For the Aim 4 literature review, we used seven publications on ISCBPs as an initial set of “seed articles” for the identification of IS competencies—this included a seminal review on training needs for ISCBPs (Chambers et al., 2017) and publications on ISCBPs referenced therein as the primary articles for that review (Gonzales et al., 2012; Meissner et al., 2013; Norton, 2014; Padek et al., 2015; Proctor et al., 2013; Stamatakis et al., 2013). Using a snowball search method, we completed a reference review and citation review in SCOPUS to identify any supplementary articles either cited by one of the seven seed articles or referenced in these seed articles—this was Step 1 of the literature review.

We then completed the second step of reference review and citation review on the articles captured in Step 1 of the SCOPUS search that were published ISCBPs that cited the use of competencies in curriculum development. For the purposes of this project, we operationalized the definition of “competencies” to include specific skills, learning objectives, or broad domains related to IS. Once the search outcomes were consolidated and de-duplicated, one reviewer (EG) completed a title/abstract screen, and two reviewers (SJ, EG) completed the full-text review. Discrepancies between reviewers were resolved by consensus. Competencies were then extracted from the included articles. For the third and final step of the Aim 4 process to identify distinct IS competencies in use, we identified other novel IS competencies in prevailing use by other, non-published ISCBPs from the Aim 3 survey results—in addition to those extracted from the SCOPUS literature search.

### Methods for Aim 4 Modified Delphi Process to Develop Consensus on Updated IS Competencies

Two members of the research team (SJ, AP) mapped the extracted competencies from the literature review and the environmental scan to the current gold standard IS competencies (Padek et al., 2015). If an extracted competency did not match a current IS competency (Padek et al., 2015), it was considered to be unique and novel. Discrepancies between reviewers were resolved by consensus. The list was edited by the core research team based on redundancy, clarity of meaning, and relevancy to IS research to a final list of 10 novel IS competencies.

We identified experts in the field of IS to determine if (1) the 10 novel competencies should be added to the current list (Padek et al., 2015) and (2) the hierarchical training levels of the novel competencies. To do this, we employed the use of a modified Delphi process and card sort approach in a web-based survey technology platform (Qualtrics©). Based upon our team's prior experience with IS competency development and refinement (Padek et al., 2015), we sought to include previous participants from ISCBPs and those with at least a base knowledge of the field, in order to obtain more accurate results. The experts identified (*n* = 258) consisted of faculty from the University of Colorado Adult and Child Center for Health Outcomes Research & Delivery Service (ACCORDS) program (*n* = 16), faculty and fellows from the 2010–2018 Implementation Research Institute (IRI, R25MH080916) training program (*n* = 96), the 2014–2017 Mentored Training for Dissemination and Implementation Research in Cancer (MT-DIRC, R25CA171994) training program (*n* = 52), and the Washington University Dissemination and Implementation Research (WUNDIR) network (*n* = 94).

After consenting to complete the survey, we asked respondents to rate each novel IS competency on importance to add to the current IS competency list (i.e., *How important do you feel it is to add the following competency to the current IS competency list* [Padek et al., 2015], from 1 “not at all important” to 5 “extremely important”). Participants were also asked to place each competency statement into the column that best expressed the skill level needed to address that competency. The columns were labeled “Beginner,” “Intermediate,” and “Advanced.” These learning levels were not defined, in order to allow participants to self-define the categories and exclude any unintentional bias from the research team on these definitions. This step mimicked the card sort process used to develop the current IS competencies (Padek et al., 2015). After this activity, participants were asked to denote their (1) discipline (public health, health sciences, social work, etc.); (2) level of expertise in the field of IS research (beginner, intermediate, advanced); and (3) country of residence.

### Data Analysis for IS Competency Update

We used descriptive statistics to analyze the results of the expert panel survey. Mean score and standard deviation were calculated for each competency based upon rated level of importance. Novel competencies were eliminated if <50% of experts rated the competency as “very” or “extremely important” to add to the current IS competency list. For the card sort portion of the survey, statements placed within the learning level groups (beginning, intermediate, or advanced) were coded as “1, 2, or 3,” respectively. Competencies were then sorted in ascending order and divided into tertiles based upon the distribution of scores. Competencies that fell within the first tertile were categorized as beginner, the second tertile as intermediate, and the third as advanced. Minor adjustments between the learning level groups were made if a particular competency had a higher distribution of scores in a different experience level than the placement of the competency by its mean score. For example, if a competency fell in the second tertile (i.e., intermediate) but had a higher frequency of scores in the beginner level, the team would shift the competency to the beginner level to reflect the majority of responses.

## Results

### Survey Participants

The Aim 1–3 internet search strategy for ISCBPs yielded 74 ISCBPs who were contacted thrice between July and August 2020. Among these, 46 eligible programs responded to the survey request (62% response rate).

### Survey Findings for ISCBP Contextual Factors (Aim 1), Sustainment (Aim 2), and Rigor (Aim 3)

In terms of Aim 1 findings, the survey revealed programs within each of our four specified ISCBP categories: (1) academic graduate programs: graduate degree-granting programs (*n* = 6) and graduate certificate programs (*n* = 6), (2) mentored fellowships: K12 training programs (*n* = 9) or postdoctoral fellowships (*n* = 5), (3) national training programs (*n* = 8), and (4) other training (*n* = 12), as shown in Tables 1–4. Degree-granting programs often represented an IS focus or concentration as a part of a broader research training (e.g., master's in public health programs) rather than an IS degree specifically. Accordingly, the number of courses on IS methods tended to be similar across the graduate degree-granting programs and the graduate certificate programs. Degree- or certificate-granting programs typically ranged from 3 to 5 graduate-level courses expected to be completed over ∼2–3 years (Table 1). Among the *n* = 34 graduate programs, mentored fellowship programs, and national training programs, 100% reported IS researchers as a target trainee type; seven of these programs (21%) also reported enrolling implementers/implementation practitioners, program evaluators or scientists conducting quality improvement (QI) research. In contrast, among the *n* = 12 other IS programs (Table 4), *n* = 11 (92%) reported targeting implementers/evaluators/QI researchers as trainees. A distinguishing characteristic of graduate programs, K12 training programs, and fellowships versus national and other training programs concerned the need for the former to require a formal student and/or employee affiliation with the host institution.

**Table 1.** High-Level Overview of ISCBPs Yielding Graduate Degrees or Certificates.

Degree-granting programs—program name (country) year founded	Program area of focus	Program format (duration)	Target audience and tuition costs for trainees
University of Heidelberg (Germany) 2015	Core/General IS research methods, Qualitative and quantitative methods; Specific IS competencies: yes	5+ courses; 17+ credit hours (25–35 months)	MSc students in Health Services and Implementation Science (tuition: yes)
World Health Organization (WHO) TDR postgraduate training (multiple lower-middle income countries) 2015	Content focus on infectious diseases of poverty (e.g., tuberculosis, HIV); Specific IS competencies: yes	5+ courses; 17+ credit hours (13–23 months)	MSc students; seek trainees from LMIC countries (tuition: no)
University of Washington (USA) 2012	Core/General IS research methods, In-depth Implementation Science Methods; Specific IS competencies: yes	5+ courses; 17+ credit hours (3+ years)	PhD students in Global Health Metrics and Implementation Science (tuition: yes)
George Washington University (USA) 2016	Core/General IS research methods, Mixed-methods, pragmatic randomized trial program theory; Specific IS competencies: yes	5+ courses; 17+ credit hours (3+ years)	PhD students in Translational Health Sciences (tuition: yes)
Johns Hopkins University (USA) 2015	Core/General IS research methods; Specific IS competencies: yes	5+ courses; 17+ credit hours (3+ years)	DrPH students—IS concentration/track (tuition: yes)
Yale University Center for Methods in Implementation Science (USA) 2018	Core/General IS research methods, learning health care systems, development of advanced qualitative, quantitative and health economic methods; Specific IS competencies: yes	5+ courses; 17+ credit hours (3+ years)	MSc or PhD students—IS concentration/track (tuition: no for PhDs; yes for some MSc)
Certificate programs—program name (country) year founded	Program area of focus	Program format (duration)	Target audience and tuition costs for trainees
Indiana University Graduate Certificate in Innovation and Implementation Science (USA) 2015	Core/General IS research methods, Agile Innovation process, Agile Implementation Process and Agile Diffusion Process; Specific IS competencies: yes	5+ courses; 13–17 credit hours	Online delivery Early-/mid-career faculty researchers; Implementers/Evaluators; QI leaders (tuition: yes)
University of California San Francisco Implementation Science Certificate Program (USA) 2008	Core/General IS research methods; Specific IS competencies: yes	5+ courses	Online delivery; MSc or PhD students; Postdoc fellows; Early-/mid-career faculty researchers; Implementers/Evaluators; QI leaders (tuition: yes)
University of Colorado Graduate Certificate in Dissemination and Implementation Science Research (USA) 2019	Core/General IS research methods; Specific IS competencies: yes	5+ courses 12 credit hours (18–36 months)	Online delivery; MSc or PhD students; Postdoc fellows; Early-/mid-career faculty researchers; Implementers/Evaluators (tuition: yes)
University of Florida Graduate Certificate in Implementation Science (USA) 2018	Core/General IS research methods;	3–4 courses/12 or less credit hours	Hybrid in-person/online; MSc students; Early-career faculty researchers; Implementers/Evaluators; QI leaders (tuition: yes)
University of Maryland Implementation and Dissemination Science Certificate (USA) 2018	Core/General IS research methods, Global Health Perspective; Specific IS competencies: yes	3–4 courses/12 or less credit hours	Online delivery; MSc or PhD students; Postdoc fellows (tuition: yes) Early-/mid-career faculty researchers; (tuition: yes) Implementers/Evaluators; QI leaders (tuition: yes)
Washington University in St. Louis, Graduate Certificate in Dissemination and Implementation Science (USA) 2020	General IS research methods; Specific IS competencies: yes	5+ courses; 16+ credit hours (25–35 months)	Hybrid in-person/online; MSCI or PhD students; Postdoc fellows; Early-/mid-career faculty researchers (tuition: yes)

*Note.* IS = implementation science; ISCBP = Implementation Science Capacity Building Program; QI = quality improvement; USA = United States of America; Category of “Implementers/Evaluators” was defined for respondents as “Program implementers/evaluators”; Inclusion criteria: 3+ courses in IS methods for a graduate certificate or degree-granting program

**Table 2.** High-Level Overview of ISCBP Postdoctoral Fellowships and K12 Programs.

Program name (country) year founded	Program area of focus	Program format (duration)	Target audience and tuition costs for trainees
Postdoctoral fellowships			
Columbia University Global Mental Health Fellowship (USA) 2012	Core/General IS research methods, Mental Health and Substance Use	Mentored 3-year fellowship; No IS coursework required	Postdoctoral students (tuition: no)
U.S. Department of Veterans Affairs (USA) 2019	Rural Mental Health	Mentored 2-year fellowship; No IS coursework required	Postdoctoral students (tuition: no)
University of California Berkeley Postdoctoral Fellowship in Health Equity and Implementation Science (USA) 2019	Core/General IS research methods, IS research methods to address racial/ethnic disparities; Specific IS competencies: Yes	Mentored 2- to 3-year fellowship; No IS coursework required	Postdoctoral students (tuition: no)
University of California San Francisco Research in Implementation Science for Equity (RISE) (USA) 2014	Core/General IS research methods	Mentored 2-year fellowship; No IS coursework required	Postdoctoral students, Early-career faculty researchers (tuition: no)
University of Pennsylvania Postdoctoral Fellowship in Implementation Research in Mental Health and Substance Abuse Treatment (USA) 2016	Core/General IS research methods, Mental Health, Community Partnership; Specific IS competencies: Yes	Mentored 2-year fellowship; 1–2 courses/12 or less credit hours	Postdoctoral students (tuition: no)
NHLBI-funded K12 programs^a^ (2017–2022)			
Buffalo University K12 Faculty Scholar Program in Implementation Science (USA) 2017–2022 ^a^	Core/General IS research methods;	Mentored 2-year fellowship; No IS coursework required	Early-/mid-career faculty researchers (tuition: no)
Duke University NHLBI K12: Dissemination and Implementation Science in Cardiovascular Outcomes (DISCO) (USA) 2017–2022 ^a^	Core/General IS research methods; Specific IS competencies: Yes	Mentored 2-year fellowship; No IS coursework required	Early-career faculty researchers (tuition: no)
University of California San Francisco IMPACT K12 (IMplementation Science for Pulmonary And Cardiac research Training) (USA) 2017–2022 ^a^	Core/General IS research methods, Heart and Lung Diseases	Mentored 2- to 3-year fellowship; No IS coursework required	Early-career faculty researchers (tuition: no)
University of Colorado IMPACT NHLBI K12 Training Program (USA) 2017–2022 ^a^	Core/General IS research methods, Heart, Lung, and Blood diseases, Pragmatic Research Methods; Specific IS competencies: Yes	Mentored 2-year fellowship; 3–4 IS courses required (2017–2019); IS graduate certificate required (2019–2022)	Early-career faculty researchers (tuition: no)
University of Massachusetts Consortium for Cardiopulmonary Implementation Science Scholars (USA) 2017–2022 ^a^	Cardiopulmonary Health; Specific IS competencies: Yes	Mentored 2-year fellowship; No IS coursework required	Early-career faculty researchers (tuition: no)
University of Washington Implementation Science Training Program (USA) 2017–2022^a^	General IS research methods, critical care, pulmonary care, palliative care; Specific IS competencies: Yes	3 years	Early-career faculty researchers (tuition: no)
Vanderbilt University Scholars in T4 Translational Research (V-STTaR) (USA) 2017–2022 ^a^	Heart, Lung, Blood, and Sleep disorders; Specific IS competencies: Yes	Mentored 2- to 3-year fellowship; 1–2 courses/12 or less credit hours	Early-career faculty researchers (tuition: no)
Washington University K12 Mentored Training in Implementation Science Program (MTIS) (USA) 2017–2022 ^a^	Core/General IS research methods, Heart, Lung, Blood, and Sleep Disorders; Specific IS competencies: Yes	Mentored 2-year fellowship; No IS coursework required	Early-/mid-career faculty researchers (tuition: no)
Yale University Scholars in Implementation Science (YSIS) K12 (USA) 2017–2022 ^a^	Core/General IS research methods, Heart, Lung, Blood and Sleep Disorders; Specific IS competencies: Yes	Mentored 2- to 3-year fellowship; 3–4 IS courses required	Postdoctoral students, Early-career faculty researchers (tuition: no)

*Not*e. IS = implementation science; ISCBP = Implementation Science Capacity Building Program; USA = United States of America; Inclusion criteria: mentored training in IS methods.

^a^NHLBI K12 funding supported several IS training programs from 2017 to 2022.

**Table 3.** High-Level Overview of ISCBP National Programs.

Degree-granting programs—program name (country) year founded	Program area of focus	Program format (duration)	Target audience for trainees
Mentored Training for Dissemination and Implementation Research in Cancer (MT-DIRC) (USA) 2014–2018	Broad introduction to IS methods; Cancer prevention and control focus; Specific IS competencies: Yes	Weeklong training + mentoring for 24 months	Early- to mid-career researchers new to IS methods
Implementation Research Institute (IRI) (USA) 2010	Core/General IS research methods, Mental Health, Substance Use, VA, Children's mental health policy; Specific IS competencies: Yes	Weeklong training + mentoring for 24 months	Early-career researchers new to IS methods
Training Institute in Dissemination and Implementation Research in Health (TIDIRH) (USA) 2011–2019	Any content area; Specific IS competencies: Yes	Weeklong training	All researchers new to IS methods
Institute for Implementation Scholars (IS-2) (USA) 2020	Existing IS researchers seeking to further develop expertise; Specific IS competencies: Yes	2-year period of training and mentoring	Early- and mid-career faculty researchers
King’s College London Implementation Science Masterclass (UK) 2014	Core/General IS research methods, Theoretical frameworks and models, implementation outcomes and strategies, economic analysis, designing and implementing interventions, sustainability, patient and public involvement, dissemination; Specific IS competencies: Yes	2-day intensive; no IS coursework required	MS and PhD students, Postdoctoral students, Early- and mid-career faculty researchers, Program implementers/evaluators, QI leaders, Patients and the public, health (clinical and allied) and social care professionals
National Cancer Institute Training Institute for Dissemination and Implementation Research in Cancer (TIDIRC) (USA) 2018	Broad introduction to IS methods; Cancer prevention and control focus; Specific IS competencies: Yes	6-month period of training and facilitated completion of one online IS course	Postdoctoral students, Early- and mid-career faculty researchers, Practitioners, Senior Career
Training Institute in Dissemination and Implementation Research in Health (TIDIRH Australia) (AUS) 2019	Broad introduction to IS method; Specific IS competencies: Yes	6 months; no IS coursework required	PhD and Postdoctoral students, Early- and mid-career faculty researchers, Program implementers/evaluators and QI leaders
HIV, Infectious Disease and Global Health Implementation Research Institute (USA) 2021	Core/General IS research methods, HIV, infectious diseases, global health. Specific IS competencies: Yes	2-year period of training and mentoring	Early-career infectious disease, HIV, and global health researchers (PhDs/MDs and equivalent) at postdoctoral or junior faculty level. US and non-US citizens

*Not*e. AUS = Australia; IS = implementation science; ISCBP = Implementation Science Capacity Building Program; HIV = human immunodeficiency virus; QI = quality improvement; UK = United Kingdom; USA = United States of America; Category of “Implementers/Evaluators” was defined for respondents as “Program implementers/evaluators”; Inclusion criteria: multi-day intensive national training.

**Table 4.** High-Level Overview of Other Intensive ISCBPs.

Program name (country) year founded	Program area of focus	Program format (duration)	Target audience for trainees
Indiana University Agile Implementation Bootcamp (USA) 2017	Agile Implementation Process; Specific IS competencies: Yes	<6 months	MS and PhD students, Postdoctoral students, Early- and mid-career faculty researchers, program implementers/evaluators, QI leaders
Sick Kids Knowledge Translation Professional Certificate (Canada) 2010	General IS research methods, IS methods in practice (e.g., planning, evaluating, stakeholder engagement, use of social media, interacting with the policy community, etc.)	5 days	Program implementers/evaluators in clinical or public health settings
Sick Kids Planning for Implementation Practice (PIP) (Canada) 2019	General IS research methods, Participants are taught how to use an evidence-based implementation planning framework to plan for their implementation endeavor	2 days	Program implementers/evaluators in clinical or public health settings
Sick Kids Specialist Knowledge Translation Training (Canada) 2004	General IS research methods (planning, evaluating, IS strategies, plain language communication)	3 days	Early-career faculty researchers, mid-career faculty researchers, QI leaders, Other
University of Arkansas Implementation Science Scholars Program (USA) 2020	General IS research methods, includes focus on the overlap between QI, improvement science, and IS	2 years	QI leaders/officers
University of California San Francisco Implementation Science Short Course (USA)	General IS research methods	2 days	Postdoctoral students, Early-career faculty researchers, mid-career faculty researchers, program implementers/evaluators in clinical or public health settings, Quality Improvement program leaders/officers
University of Massachusetts Prevention and Control of Cancer: Training for Change in Individuals and Systems (USA) 2014	Cancer Prevention and Control; Specific IS competencies: Yes	2 years	Doctoral students, Postdoctoral students
University of Missouri-Kansas City Training in Implementation: Actionable Research Approaches (TIARA) (USA) 2018	General IS research methods; Specific IS competencies: Yes	<6 months	MS, PhD, and Postdoctoral students, Early- and mid-career faculty researchers, Program implementers/evaluators and QI program leaders/officers
University of South Florida Institute for Translational Research Education in Adolescent Drug Abuse (USA) 2012	General IS research methods, Adolescent behavioral health including alcohol use/abuse, drug use/abuse, mental disorders, and at-risk populations (e.g., Native American youth and families, foster care youth)	1 year	MS and PhD students, Program implementers/evaluators, Clinicians, EBP developers
University of Washington Fundamentals of Implementation Science Online Course (USA) 2015	General IS research methods	<6 months	MS students, Program implementers/evaluators, QI program leaders/officers
University of Washington Implementation Science Intensive Summer Institute (USA) 2014	General IS research methods, There are 2 tracks: intro to IS and advanced IS methods	<6 months	MS/PhD students, Postdoctoral students, Early-career faculty researchers, Program implementers/evaluators
World Health Organization (WHO) TDR Massive Open Online Course on Implementation Research with a focus on Infectious Diseases of Poverty (Switzerland) 2016	Illustration of IS concepts with examples from Lower Middle Income countries; Specific IS competencies: Yes	<6 months	Early-career faculty researchers, mid-career faculty researchers, program implementers/evaluators

*Note.* IS = implementation science; ISCBP = Implementation Science Capacity Building Program; Inclusion criteria: multi-day intensive training.

In terms of Aim 2 findings related to sustainment, the vast majority of ISCBPs (79%) reported ongoing annual administrative costs to support the director(s) and/or program administrators. Some programs supported these administrative costs through grant funding, but others used alternative funding approaches, including institutional support, donor support, and student tuition/fees. Typically, student tuition/fees were not sufficient to fully cover an ISCBPs' administrative costs. A smaller number of ISCBPs also supported trainee fees (46%) and teaching faculty stipends (26%), respectively.

In terms of Aim 3 findings related to rigor and IS competencies used, 74% of all ISCBP programs (*n* = 34) reported training in general/core IS methods (e.g., IS frameworks, implementation outcomes, and measures), and often reported training in other specific IS methods (e.g., patient/public involvement in research; economic analysis, learning health systems). The research focus often aligned with the sponsor's priorities, such as the nine mentored K12 programs funded by the National Heart, Lung, and Blood Institute (NHLBI) from 2017 to 2022. Global health research was also common (*n* = 4 programs). According to program leader survey respondents, IS competencies guided the curriculum among the majority of national ISCBPs (100%), graduate programs (92%), and K12 training programs (78%). However, only 40% of the postdoctoral fellowships (Table 2) and 33% of the other trainings (Table 4) reported the use of IS competencies to inform their curriculum.

### Aim 4 Revised IS Competencies

To develop the revised Aim 4 IS competencies, we identified 10 novel IS competencies from the 401 potential unique IS competencies identified in our three-step search approach. We derived these competencies in this way: the first two steps of our SCOPUS literature review search strategy yielded 5,029 articles, of which 16 articles met the eligibility criteria. The third step was to review the Aim 3 IS competencies submitted ISCBPs: this yielded 17 unique additional lists of competencies (or articles) for our consideration. From the combined 33 articles/lists, we extracted 401 discrete competencies. After comparing these 401 competencies to the current gold standard for IS competencies (Padek et al., 2015), we identified 10 novel competencies to be included in the modified Delphi process with our expert panel.

Among the 258 IS researchers and/or ISCBP leaders invited to participate in the modified Delphi process, 118 individuals completed the expert panel survey (response rate = 46%). The most common disciplines reported by respondents included public health (33%), health sciences (21%), health systems/health service administration (13%), and Psychology (12%). Just over half of the respondents (*n* = 61) described there IS expertise as “Intermediate.” Thirty-nine respondents described their expertise as “Advanced” (33%), and 19 respondents (17%) described their expertise as “Beginner.” Most respondents were from the United States (>95%).

The mean scores for importance for the 10 novel competencies ranged from 4.31 to 3.34 (Table 5). Eight out of 10 novel competencies were rated as “very” (score = 4) or “extremely important” (score = 5) by more than half of the experts and were incorporated into the updated IS competency list. Two out of those eight competencies (25%) fell into the “Advanced” category, with the remaining competencies rated as “Beginner” (38%) and “Intermediate” (38%; Table 6).

**Table 5.** Novel/Emerging Competency Descriptions and Rankings.

	Mean importance	*SD*	Count of respondents rated competency very or extremely important, *N* (%)
Design strategies to address the multi-level influences of health inequities as it relates to the implementation of an evidence-based intervention	4.31	0.84	100 (84.7)
Integrate strategies within D&I research to facilitate meaningful stakeholder engagement (e.g., shared power, shared decision-making, co-learning)	4.27	0.78	100 (84.7)
Operationalize hybrid effectiveness-implementation designs when appropriate to accelerate the implementation of evidence-based interventions in real-world settings	4.14	0.87	94 (79.7)
Develop strategies to promote equity in resource distribution across all external research partners, including community partners or other external organizations and the researcher's institution	4.00	0.94	80 (67.8)
Summarize the importance of ethically and culturally competent clinical and community-based research in D&I science	3.98	0.92	85 (72.0)
Develop and assess processes and outcomes that support iterative cycles of implementation and bidirectional flow of information (e.g., learning health systems)	3.90	0.89	79 (66.9)
Characterize process models that support iterative cycles of implementation and adaptation based on learning	3.75	0.99	73 (61.9)
Examine the importance of rapid research to advance D&I science concepts and directions	3.70	1.00	65 (55.1)
Apply theory and strategies from team science to promote team effectiveness in D&I research	3.47	0.95	55 (46.6)
Apply systems science and systems modeling approaches in D&I research	3.34	0.99	53 (44.9)

*Note*. D&I = dissemination and implementation; *SD* = standard deviation.

**Table 6.** Updated Competencies by Domain and Level of Trainee Expertise Needed to Attain.

D&I competency	D&I expertise^a^
Section A: definition, background, and rationale	
Define and communicate dissemination and implementation (D&I) research terminology	B
Define what is and what is not D&I research	B
Differentiate between D&I research and other related areas, such as efficacy research and effectiveness research	B
Identify the potential impact of disseminating, implementing, and sustaining effective interventions, including assessments of equity and representativeness	B^b^
Describe the range of expertise needed to conduct D&I research (e.g., mixed-methods experience, economic, organizational, policy, clinical)	B
Examine the importance of rapid research to advance D&I science concepts and directions	B^c^
Determine which evidence-based interventions are worth disseminating and implementing	I
Assess, describe, and quantify (where possible) the context for effective D&I (setting characteristics, culture, capacity, and readiness)	I
Identify existing gaps in D&I research	I
Identify the potential impact of scaling down (aka de-implementing) an ineffective but often used intervention	I
Formulate methods to address barriers and facilitators of D&I research	I^b^
Section B: theory and approaches	
Describe a range of D&I strategies, models, and frameworks	B
Design strategies to address the multi-level influences of health inequities as it relates to the implementation of an evidence-based intervention	I^c^
Identify appropriate conceptual models, frameworks, or program logic for D&I change	I
Identify core elements (effective ingredients) of effective interventions, and recognize risks of making modifications to these	I
Describe a process for designing for dissemination (planning for adoption, implementation, and sustainability during the intervention development stage)	I
Describe the relationships between various organizational dimensions (e.g., climate, culture) and D&I research	I
Characterize process models that support iterative cycles of implementation and adaptation based on learning	I^c^
Explain how knowledge from disciplines outside of health (e.g., business, marketing, and engineering) can help inform further transdisciplinary efforts in D&I research	I
Identify and articulate the interplay between policy and organizational processes in D&I	I
Section C: design and analysis	
Describe the core components of external validity and their relevance to D&I research	B
Identify common D&I measures and analytic strategies relevant to your research question(s)	B
Identify and measure outcomes that matter to stakeholders, adopters, and implementers	I
Describe the application and integration of mixed-method (quantitative and qualitative) approaches in D&I research	I
Apply common D&I measures and analytic strategies relevant for your research question(s) within your model/framework	I
Identify possible methods to address external validity in study design reporting and implementation	I
List the potential roles of mediators and moderators in a D&I study	I
Identify and articulate the trade-offs between a variety of different study designs for D&I research	I
Describe how to frame and analyze the context of D&I as a complex system with interacting parts	I
Effectively integrate the concepts of sustainability/sustainment and the rationale behind them in D&I study design	I
Describe gaps in D&I measurement and critically evaluate how to fill them	A
Operationalize hybrid effectiveness-implementation designs when appropriate to accelerate the implementation of evidence-based interventions in real-world settings	A^c^
Develop and assess processes and outcomes that support iterative cycles of implementation and bidirectional flow of information (e.g., learning health systems)	A^c^
Effectively explain and incorporate concepts of de-adoption and de-implementation into D&I study design	A
Incorporate methods of economic evaluation (e.g., implementation costs, cost-effectiveness) in D&I study design	A
Evaluate and refine innovative scale-up and spread methods (e.g., technical assistance, interactive systems, novel incentives, and “pull” strategies)	A
Section D: practice-based considerations	
Describe the importance of incorporating the perspectives of different stakeholder groups (e.g., patient/family, employers, payers, healthcare settings, public organizations, community, and policy makers)	B
Summarize the importance of ethically and culturally competent clinical and community-based research in D&I science	B^c^
Describe the concept and measurement of fidelity	B
Articulate the strengths and weaknesses of participatory research in D&I research	B
Integrate strategies within D&I research to facilitate meaningful stakeholder engagement (e.g., shared power, shared decision-making, co-learning)	B^c^
Determine when engagement in participatory research is appropriate with D&I research	I
Describe the appropriate process for eliciting input from community-based practitioners for adapting an intervention	I
Identify and apply techniques for stakeholder analysis and engagement when implementing evidence-based practices	I
Identify a process for adapting an intervention and implementation strategy prior to and during implementation	I^b^
Describe how adaptations will be documented throughout the D&I research project	I^b^
Explain how to maintain fidelity of original interventions during the adaption process	I
Identify sites to participate in D&I studies, and negotiate or provide incentives to secure their involvement	I
Identify and develop sustainable partnerships for D&I research	I
Develop strategies to promote equity in resource distribution across all external research partners, including community partners or other external organizations and the researcher's institution	I^c^
Describe how to measure successful partnerships for D&I research	I
Use evidence to evaluate and adapt D&I strategies for specific populations, settings, contexts, resources, and/or capacities	A

*Note.* D&I = dissemination and implementation.

^a^ Expertise levels: B  =  beginner; I  =  intermediate; A  =  advanced; ^b^ Updated competency; ^c^ Novel/emerging competency.

We categorized the eight novel/emerging IS competencies within the original four categories of IS competencies developed by Padek et al.: 1. Definition, Background, and Rationale; 2. Theory and Approaches; 3. Design and Analysis; 4. Practice-based considerations (Table 6). In terms of thematic commonalities of the 10 novel competencies, three explicitly address the intersection of health equity and IS: (a) Design strategies to address the multi-level influences of health inequities as it relates to the implementation of an evidence-based intervention; (b) Develop strategies to promote equity in resource distribution across all external research partners, including community partners or other external organizations and the researcher's institution; (c) Summarize the importance of ethically and culturally competent clinical and community-based research in Dissemination and Implementation science. Four of the novel competency statements relate to enhancing the impact of translational research (Peek et al., 2014) by making implementation more rapid and/or recursive, including the conduct of iterative cycles of implementation and shared data use approaches such as those found in Learning Health Systems (Bennett et al., 2020; Damschroder et al., 2021; Kilbourne et al., 2020). These novel competencies related to making implementation more rapid include: (a) Operationalize hybrid effectiveness-implementation designs when appropriate to accelerate the implementation of evidence-based interventions in real-world settings; (b) Develop and assess processes and outcomes that support iterative cycles of implementation and bidirectional flow of information (e.g., learning health systems); (c) Characterize process models that support iterative cycles of implementation and adaptation based on learning; (d) Examine the importance of rapid research to advance Dissemination and Implementation science concepts and directions. Overall, we would frame these novel competencies as efforts to make IS research more rapid and inclusive, by including diverse perspectives from those in the inner setting and outer setting across all study phases, and by attending to the inclusion of cultural norms and preferences in the approaches used.

## Discussion

This multi-method study adds to the literature on ISCBPs in terms of their focus areas and characteristics, the rigor and IS competencies addressed, and factors related to their sustainment. We found that current ISCBPs train learners across varying career stages in diverse focus areas, in line with expert recommendations (Leppin et al., 2021; Proctor & Chambers, 2017). Related to sustainment of ISCBPs, we found that nearly 80% of ISCBPs reported ongoing annual administrative costs, and alternative grant funding or institutional funding sources in addition to student tuition/fees were recommended to maintain solvent programs. Related to the rigor of IS training, we found that while >70% of respondent ISCBPs addressed core/general IS methods, targeting IS competencies was less consistent. Specifically, ≤40% of non-graduate, non-fellowship, and postdoctoral fellowship ISCBPs reported addressing specific IS competencies, while over 90% of graduate and national ISCBPs targeted IS competencies. In addition, our expert panel came to consensus on a set of eight highly important IS competencies that are unique additions to the current gold standard for IS competencies (Padek et al., 2015). Thematically, these eight novel competencies represent a need to provide training at the intersection of IS and equity, as well as the need for more rapid translation of EBPPs.

The updated set of IS competencies is valuable for ISCBPs to develop and/or expand their curriculum, particularly for content related to the intersection of health equity and IS, and the rapidity of translating evidence into practice. These findings are also complementary with the domains of implementation competencies recently identified for implementation practitioners applying IS—as distinct from competencies for researchers (Leppin et al., 2021). Areas of overlap include the development of competencies for implementation practitioners to improve the rapidity of implementation by “diagnosing” contextual barriers and facilitators to implementation, and “treating” those contextual factors by developing and deploying appropriate implementation strategies (Leppin et al., 2021).

In terms of sustainment, we found that the vast majority of ISCBPs require ongoing funding from grants or institutional funds, in addition to trainee tuition/fees, in order to support their program administrators, and a smaller number also provide stipends to instructors. These findings relate to recommendations in a recent review by Proctor and Chambers to consider the *sustainability* of ISCBPs: to ensure funding sources and human capital are available to meet the training needs (Proctor & Chambers, 2017). The relevant staffing and administrative requirements for launching and sustaining an IS training program are important to consider at the outset. Most recently, Proctor and colleagues discussed the need for developing market viability considerations in the field of IS that are relevant to ISCBPs, a skill set less commonly obtained through traditional public health and health services research training (Proctor et al., 2021). One program specifically noted requiring 20% full-time equivalent (FTE) of effort for Graduate Certificate Program Directors and 25% FTE of effort for an administrative assistant, but noted this would be much higher—up to 50% FTE for the Director and 100% FTE for an administrative assistant in the absence of existing graduate school and NIH-funded Clinical Translational Science Agency (CTSA) resources leveraged to support that program. Another recommendation was to consider how to appropriately compensate IS research faculty for teaching, as compared to their compensation for serving as a co-investigator on grants. Based on the literature and the findings of this multi-method scoping review, survey, and modified Delphi process, factors relevant to ISCBP sustainment include:

1. Ability to differentiate from other ISCBPs to meet the needs of specific subsets of trainees, such as relevant local experts who can teach specific subsets of IS competencies or train individuals to apply IS methods to a specific content area (e.g., global health, cardiopulmonary disease, mental health);
2. Adequate capacity for the proposed faculty/mentors, as the resources of faculty availability/time will dictate the number of courses taught and the number of trainees that the program can mentor—for this reason, some institutions may start by offering 1–2 IS courses rather than launching a full ISCBP Graduate Certificate or IS concentration, as the latter typically includes 3 or more IS-focused courses;
3. Appropriate financial sponsorship/support—availability (or not) of external training grant support, or internal support in the form of administrative sponsorship by a Dean/Department chair, a local NIH-funded CTSA, and/or a local doctoral or master's level graduate training program that may wish to offer an IS concentration (or Certificate) as part of their existing program of training—these types of support are important as student fees will not typically cover the administrative costs of delivering a program;
4. Sufficient infrastructure to provide distance learning (online platform) and/or in-person learning;
5. Trainee demand for the type of training offered based on the above four elements

This review has several strengths and limitations. Its strengths include the robust, multi-method approach to identify ISCBP characteristics and competencies through the published academic literature and internet, as well as using snowball sampling and surveys of ISCBP leaders. Related to our inclusion criteria for graduate programs to offer at least three IS courses, this is not a comprehensive list of graduate programs with IS training concentrations, and also excludes individual graduate courses. Limitations of our multi-method literature search include the potential to miss publications of international ISCBPs by restricting our literature search to English-language publications, and the possibility to overlook publications of IS competencies that were not described in the title/abstract. Furthermore, given the large number of articles to screen, we only had capacity for one author to review the titles/abstracts, but did include two authors to review the full articles for possible IS competencies. The updated competency list was compiled based on a modified Delphi process with a snowball sample of international IS experts which may underrepresent the perspective of implementation practitioners. In addition, this process was undertaken prior to a recent publication by Schultes and colleagues on clusters of general IS competencies (e.g., knowledge and skills of IS, setting, program evaluation, and research methodology), so their recommended IS competencies were not incorporated into our modified Delphi process (Schultes et al., 2021). However, we do not find these clusters by Schultes et al. to be fundamentally distinct from the current Padek et al. competencies that we used (Schultes et al., 2021). Finally, Proctor and Chambers emphasized the need to provide support to alumni of IS training programs (Proctor & Chambers, 2017) and the need to provide mentored application to an IS research project in addition to didactic training; however, the scope of this review was limited to the training period itself, and did not specifically address the use of mentored research projects.

## Conclusions and Recommendations

Our findings are a snapshot of the evolving field of IS training in a phase where the number and types of trainings continue to expand. The evolution of the field and expansion of programs warrants further attention to ISCBP rigor and sustainment, including our identification of eight novel, important IS competencies. Given our findings, we recommend the following: (1) To promote transparency: ISCBPs should report contextual characteristics of their program in line with recent recommendations (Davis & D'Lima, 2020; Proctor & Chambers, 2017) according to the examples provided in Tables 1–4, including the IS competencies addressed, program duration, course requirements, costs, and whether a program's focus is on developing IS researchers, implementation practitioners, program evaluators, QI researchers, or a combination of these; (2) To promote rigor: ISCBPs should include IS competencies consistently in the design of their curriculum, evaluate trainee self-assessment of progress toward the IS competencies targeted, and periodically revise the curriculum based on trainee gaps in progress; (3) It is not necessary for every program to address every competency. We encourage ISCBPs to select competencies with attention to the skill level of their trainees (e.g., beginner, intermediate, advanced), and with attention to the end goals of their trainees; (4) As there is a particular dearth of capacity building programs for implementation practitioners and those that exist are often siloed from current ISCBPs, we echo the call by Leppin et al. to continue to develop ISCBP models that train IS researchers and implementation practitioners together (Leppin et al., 2021), and we’ve identified several existing programs that do this (Tables 1–4); (5) We also recommend that those planning a new ISCBP anticipate the factors relevant to sustainment described above, including program differentiation from other ISCBPs, adequate faculty/mentor bandwidth, and sufficient financial support. In addition to these implications for ISCBP program leaders, there are also policy implications. As part of the program evaluation for new ISCBPs, IS journals may consider requiring reporting on as the ISCBP area of focus, trainee types accepted, and IS competencies addressed. The field may also consider developing an accreditation body to evaluate the rigor of the curriculum for ISCBPs. In sum, the future is bright for ISCBPs, but we need to continue to reflect and evolve our training goals as IS research methods also develop in this young field.
